# Conversion between the Rowland Universal Dementia Assessment Scale and Mini‐Mental State Examination test scores in majority and minority populations

**DOI:** 10.1002/brb3.3650

**Published:** 2024-09-01

**Authors:** Alfonso Delgado‐Álvarez, Maria Valles‐Salgado, Cristina Delgado‐Alonso, Jorge Matias‐Guiu, Jordi A. Matias‐Guiu

**Affiliations:** ^1^ Department of Neurology, Hospital Clinico San Carlos, San Carlos Institute for Health Research (IdiSSC) Universidad Complutense de Madrid Madrid Spain; ^2^ Department of Psychobiology & Behavioral Sciences Methods Universidad Complutense de Madrid Madrid Spain

**Keywords:** Alzheimer's disease, cross‐cultural neuropsychology, equipercentile, mild cognitive impairment

## Abstract

**Introduction:**

Despite the Rowland Universal Dementia Assessment Scale (RUDAS) having significant advantages as a cognitive screening tool, particularly for minority populations, the Mini‐Mental State Examination (MMSE) test is the most widely used test for cognitive screening in Alzheimer's disease (AD). This study aimed to develop a conversion table to predict MMSE scores from observed RUDAS scores, allowing an easy‐to‐use method to compare both screening tests.

**Methods:**

The equipercentile equating method was used to develop the conversion table using a training sample consisting of cognitively intact participants and individuals with early‐stage AD. The resulting conversion table was validated in two samples, comprising participants from majority and minority populations assessed in Spanish.

**Results:**

The conversion table demonstrated excellent reliability with intraclass correlation coefficients of.92 in both validation samples.

**Conclusion:**

This study provides a conversion table between RUDAS and MMSE scores, improving the comparability of these cognitive screening tools for use in clinical and research purposes.

## Graphical abstract

This study aimed to develop a conversion table to predict MMSE scores from observed RUDAS scores, allowing an easy‐to‐use method to compare both screening tests. The equipercentile equating method was used to develop the conversion table using a training sample consisting of cognitively intact participants and individuals with early‐stage AD. The resulting conversion table was validated in two samples, comprising participants from majority and minority populations assessed in Spanish, showing excellent reliability.

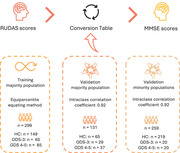

## 1 INTRODUCTION

Alzheimer's disease (AD) is the most common cause of dementia (Mayeux & Stern, 2012). In the early stages of the disease, the most prevalent symptoms are cognitive deficits, particularly episodic memory impairment (Dubois et al., 2016).

Neuropsychological assessment allows the evaluation of cognitive domains and is essential in the diagnosis of different neurodegenerative disorders, especially AD. In this regard, cognitive screening tests are commonly used as a first step during the cognitive assessment, resulting in a general overview of cognitive status. They require little training and are easy to administer and interpret, differentiating between patients with cognitive impairment and cognitively intact participants.

Although there are numerous cognitive screening tests available today (De Roeck et al., 2019), the Mini‐Mental State Examination (MMSE) test (Folstein et al., 1975) is the most widely used test for cognitive screening. However, it has been criticized for its insensitivity to detect cognitive impairments in the early stages of the disease, ceiling and floor effects, and the lack of items for the executive function domain (Devenney & Hodges, 2017).

Moreover, a significant body of literature has highlighted various limitations of the MMSE concerning cultural variables, including language, education, and ethnicity (Basic et al., 2009; Celik et al., 2022; Escobar et al., 1986; Espino et al., 2001; Jones & Gallo, 2002; Nielsen et al., 2012; Parker & Philp, 2004; Ramirez et al., 2006). In this regard, research in cross‐cultural neuropsychology has described the impact of cultural variables on test scores and has highlighted the need for cross‐cultural screening tools (Franzen et al., 2021).

The Rowland Universal Dementia Assessment Scale (RUDAS), developed from a cross‐cultural perspective, has been proposed and validated for dementia (Storey et al., 2004). RUDAS has a total score of 30 and allows the assessment of visuospatial orientation, praxis, visuoconstructive drawing, judgment, memory, and semantic fluency. Compared to the MMSE, RUDAS has proved to be less affected by language and education, following a recent meta‐analysis (Nielsen & Jørgensen, 2020). Furthermore, previous studies have shown important advantages of RUDAS against the MMSE in terms of psychometric properties and ecological characteristics and have proved its utility in different neurological diseases, including early stages of AD or Parkinson's disease with mild cognitive impairment (Celik et al., 2022; Delgado‐Álvarez, 2022; Delgado‐Álvarez et al., 2023; Goudsmit et al., 2018; Matías‐Guiu et al., 2017; Nielsen et al., 2012).

Despite the strengths of RUDAS, the MMSE is still considered a standard in various settings. In clinical settings, MMSE scores are required for acquiring cholinesterase inhibitors for AD in some countries (Matías‐Guiu et al., 2018). Meanwhile, in research settings, the MMSE allows a comparison of different cross‐sectional studies due to its high frequency of use. Conversion tables to obtain MMSE scores from RUDAS scores would thus be especially useful for those studies where RUDAS was the cognitive screening test of choice. Because RUDAS is also commonly used in the early stages of AD and in minority populations, conversion tables validated in majority populations and also in minority populations would be key.

The current study aimed to develop a conversion table to estimate MMSE scores from RUDAS in a majority population and to validate the resulting table in two samples: one majority sample and one minority sample, comprising cognitively intact participants and people with early‐stage Alzheimer's disease (pwAD).

We hypothesized that the conversion table obtained from a training sample would accurately estimate MMSE scores close to the observed MMSE scores in two validation samples from majority and minority populations, showing adequate intraclass correlation coefficients (ICC).

## 2 METHODS

### 2.1 Participants

**FIGURE 1:**
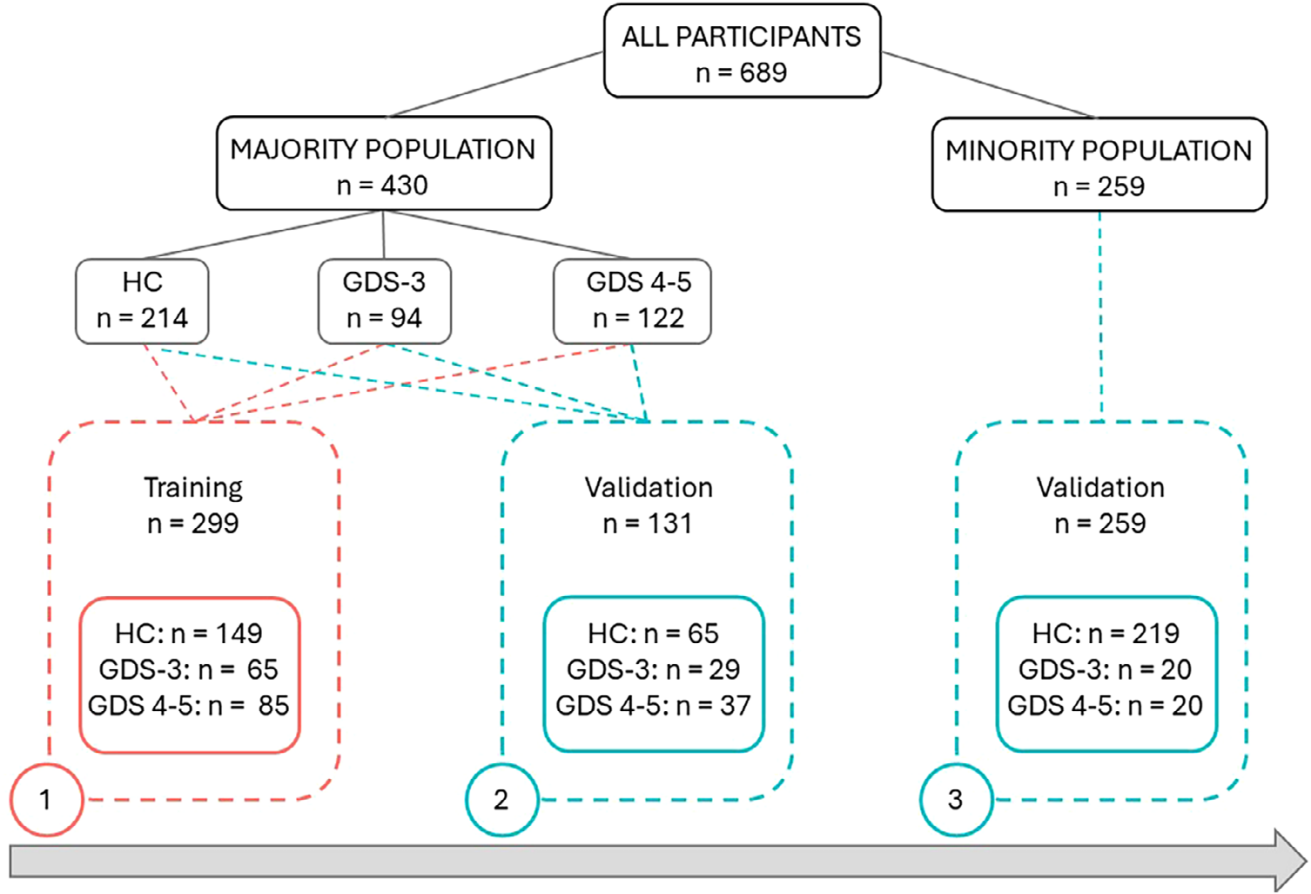
Flow chart of the study population. HC, healthy control group; GDS 3, people with Alzheimer's disease and Global Deterioration Scale 3; GDS 4–5, people with Alzheimer's disease and Global Deterioration Scale 4/5.

Six hundred and eighty‐nine participants with Spanish as their first language were recruited from the Department of Neurology at Hospital Clínico San Carlos. The sample consisted of cognitively healthy control (HC) participants (*n* = 433) and pwAD in the early stages of the disease (Global Deterioration Scale [GDS] 3–5) (*n* = 256). Participants were divided into a majority population group (*n* = 430, HC: 214, GDS 3: 94, GDS 4–5: 122) and a minority population group from different minority populations identified as Hispanic/Latino community (*n* = 259, HC: 219, GDS 3: 20, GDS 4–5: 20) (Figure 1).

Inclusion criteria for pwAD were as follows: (1) evidence from biomarkers supporting the diagnosis of AD (i.e., FDG‐PET showing hypometabolism in temporoparietal regions and/or cerebrospinal fluid with altered A‐beta 1–42, tau, and phospo‐tau levels), and (2) memory complaints in combination with Clinical Dementia Rating (CDR) scale scores of .5 and .5 in the memory box for GDS 3, CDR of 1.0 and at least .5 in the memory box for GDS 4, and CDR of 2.0 and at least .5 in the memory box for GDS 5 (Albert et al., 2011).

For the HC group, the inclusion criteria were (1) a CDR score of 0 and (2) the absence of functional impairment based on scores of 0 in the Functional Activities Questionnaire (Olazarán et al., 2005).

Exclusion criteria for all participants were (1) any physical difficulty leading to potential bias in scores (e.g., hearing or visual deficits), (2) history of neurological or psychiatric disease (e.g., epilepsy, major depression, and substance abuse), and (3) presence of medical disorders associated with cognitive impairment.

### 2.2 Procedure

This study was conducted with the approval of the Ethics Committee of the Hospital Clínico San Carlos, and all participants provided written informed consent.

Neuropsychological assessments were performed in Spanish by trained neuropsychologists in one single session. All participants were randomly assigned so that half of the participants started with the MMSE and the other half with the RUDAS.

Scores from 70% of participants comprising HC and GDS 3–5 from the majority population were randomly selected to develop the conversion table. The resulting conversion table was tested in the majority and minority validation samples (Figure 1).

### 2.3 Statistical analysis

Statistical analysis was performed using RStudio 4.3.1 and the “equate” package (Albano, 2016). Alpha was set to .05. Descriptive data are presented as mean ± standard deviation (minimum–maximum score) or frequency (percentage).

Pearson's correlation coefficient was used to explore the correlation between MMSE and RUDAS scores, interpreting *r* ≤ .29 as very low,.3–.49 as low,.5–.69 as moderate,.7–.89 as high, and ≥.90 as very high correlation. The coefficient of determination (*r* ^2^) was reported in order to describe the percentage of shared variance between both screening tests.

To obtain the equivalence between RUDAS and MMSE scores, the equipercentile equating method (Albano, 2016) was used as previously described in similar studies (Aiello et al., 2023; Fasnacht et al., 2023; Gross et al., 2019; Matías‐Guiu et al., 2018; Melikyan et al., 2021). First, both tests were linked by calculating and matching the percentiles of each screening test in the validation sample. Log‐linear smoothing available in the equate package was applied. Second, a conversion table was created to predict MMSE scores from RUDAS scores. Third, the reliability of the conversion table was tested in a validation sample from the majority population. For this purpose, the ICC was calculated between observed and obtained MMSE scores from the conversion table in the validation sample. ICC values less than .5, between .5 and .75, between .76 and .90, and greater than .90 indicated poor, moderate, good, and excellent reliability, respectively (Koo & Li, 2016). In addition, the percentage of cases that fell within ±1 and ±2 points of the true MMSE score was reported. Finally, the same validation process was replicated in the minority sample (Figure 1).

## 3 RESULTS

### 3.1 Sample characteristics and conversion table

The main demographic and clinical characteristics are shown in Table 1. The minority group represented Hispanic/Latino communities living in Spain from different countries, primarily Colombia (78.7%), followed by Peru (8%), Venezuela (4%), Ecuador (3.1%), the Dominican Republic (2.6%), Argentina (0.8%), Mexico (0.8%), Bolivia (0.4%), Chile (0.4%), Cuba (0.4%), Honduras (0.4%), and Nicaragua (0.4%).

**TABLE 1:** Demographic and clinical characteristics of the sample.

	Training (*n* = 299)	Validation—majority (*n* = 131)	Validation—minority (*n* = 259)
HC	149 (49.8%)	65 (49.6%)	219 (85%)
pwAD	150 (50.2%)	71 (50.4%)	40 (15%)
Sex, female	177 (59.2%)	74 (56.5%)	132 (51%)
Age, years	66.6 ± 17.2 (20–91)	68.1 ± 16.2 (20–93)	46.5 ± 15.4 (20–83)
Education, years	10.1 ± 5.0 (0–18)	9.3 ± 5.4 (0–18)	12.1 ± 3.8 (3–18)
RUDAS (/30)	24.9 ± 5.3 (10–30)	25 ± 5.0 (12–30)	28.5 ± 3.1 (9–30)
MMSE (/30)	26.3 ± 4.4 (12–30)	26.2 ± 4.4 (12–30)	29.0 ± 2.6 (9–30)

Abbreviations: HC, healthy control group; MMSE, Mini‐Mental State Examination; pwAD, people with Alzheimer's disease; RUDAS, Rowland Universal Dementia Assessment Scale.

The correlation between RUDAS and MMSE scores was *r* = .841, *p* < .001, *r* ^2^ = 71% in the majority population and *r* = .835, *p* < .001, *r* ^2^ = 69% in the minority population. The data for the conversion from RUDAS to MMSE scores are shown in Table 2.

**TABLE 2:** Conversion table from Rowland Universal Dementia Assessment Scale (RUDAS) to Mini‐Mental State Examination (MMSE) scores.

RUDAS	MMSE
0	0
1	2
2	4
3	5
4	6
5	7
6	8
7	9
8	10
9	11
10	12
11	13
12	14
13	15
14	17
15	18
16	19
17	20
18	21
19	22
20	23
21	24
22	25
23–24	26
25	27
26–27	28
28–29	29
30	30

### 3.2 Validation

In the majority validation sample, the ICC between observed MMSE and estimated MMSE scores was .92 (95% confidence interval [CI].89–.95, *p* < .001). The estimation fell within ±1 point of the observed MMSE scores in 66% of all cases and within ±2 points in 78% of cases. In particular, the ICC in the HC group was .77 (95% CI.61–.85, *p* < .001), and the ICC for pwAD was .87 (95% CI.79–.92, *p* < .001).

In the minority sample, the ICC between observed and estimated MMSE scores was.92 (95% CI.89–.93, *p* < .001). The estimation fell within ±1 point of the observed MMSE scores in 77% of cases and within ±2 points in 84% of cases. In the HC group, ICC was.70 (95% CI.60–.77, *p* < .001), and in pwAD was .84 (95% CI.70–.93).

## 4 DISCUSSION

The study aimed to create a conversion table between RUDAS and MMSE to bridge the gap between the utility of RUDAS as a screening test, particularly in minority populations, and the most commonly used cognitive screening test, MMSE.

Both screening tools showed a percentage of shared variance higher than 70%, supporting the equipercentile equating method (Albano, 2016). The validation studies demonstrated moderate—excellent reliability, with more than 60% of cases correctly classified with a maximum difference of one point from the observed score. Furthermore, these results were replicated in the minority sample, supporting the utility of the conversion table in Hispanic/Latino communities.

Previous studies have established the advantages of conversion tables for screening tools by comparing different versions of MMSE (Gross et al., 2019), creating conversion tables between the Montreal Cognitive Assessment test and the MMSE (Fasnacht et al., 2023; Melikyan et al., 2021), or Addenbrooke's Cognitive Examination III (ACE‐III) and MMSE (Matías‐Guiu et al., 2018). In addition, the equipercentile equating method has been applied to various neurological diseases, including AD, Parkinson's disease, and amyotrophic lateral sclerosis (Aiello et al., 2023; Matías‐Guiu et al., 2018; van Steenoven et al., 2014).

To our knowledge, no previous studies have reported RUDAS and MMSE conversion scores for early‐stage AD. This study is the first to conduct a multicultural validation, considering participants from not only majority but also minority populations, providing an easy‐to‐use conversion table.

We found higher estimated MMSE scores than observed RUDAS scores, indicating a higher level of difficulty in the RUDAS test compared to MMSE. Reliability findings were similar to previous studies in our setting in different neurological diseases using the ACE‐III. In particular, a conversion study between ACE‐III and MMSE in pwAD and cognitively HCs (Matías‐Guiu et al., 2018) showed similar ICC in the validation sample (ACE‐III study: 0.94 vs. our study: 0.91). Moreover, 60% and 81% of estimated scores in the ACE‐III study fell within ±1 score and ±2, respectively, compared to 66% and 78% in our study. Unfortunately, no previous studies in minority populations are available to compare our results.

This study has some limitations. First, we solely focused on pwAD in the early stages of the disease. Although this allowed us to obtain a conversion table for HC, GDS 3–5, this method could be potentially useful in other neurological diseases. Second, the minority validation sample included a high percentage of participants from Colombia and a smaller representation of other Latin American countries. Third, we did not include cases with differences between the language of assessment and the first language of participants, which may be relevant in multicultural settings. The conversion table provided applies only to Spanish‐speaking populations. Further studies could consider the possible impact of language on conversion score methods, extending beyond Spanish‐speaking populations. Fourth, RUDAS scores ranged from 10 to 30. For this reason, scores lower than 10 were estimated and not based on observed cases. However, scores lower than 10 in RUDAS are unusual in clinical practice, as cognitive screening is more often conducted at early to moderate stages but not in advanced dementia.

In conclusion, we developed a conversion table between RUDAS and MMSE. The resulting table was validated in two samples, including participants from majority and minority populations, demonstrating excellent reliability. The developed table could prove greatly useful in clinical and research settings to improve the comparability of both screening tests.

## AUTHOR CONTRIBUTIONS

**Alfonso Delgado‐Álvarez**: Conceptualization; visualization; data curation; formal analysis; investigation; writing—original draft; writing—review and editing; methodology. **Maria Valles‐Salgado** and **Cristina Delgado‐Alonso**: Data curation; investigation; writing—review and editing. **Jorge Matias‐Guiu**: Conceptualization; visualization; funding acquisition; investigation; supervision; writing—review and editing. **Jordi A. Matias‐Guiu**: Conceptualization; visualization; data curation; formal analysis; funding acquisition; investigation; methodology; supervision; writing—review and editing; writing—original draft.

## CONFLICT OF INTEREST STATEMENT

The authors declare no conflicts of interest.

### PEER REVIEW

The peer review history for this article is available at https://publons.com/publon/10.1002/brb3.3650

## Data Availability

The data that support the findings of this study are available from the corresponding author upon reasonable request.
